# Acid–base imbalance as a risk factor for mortality among COVID-19 hospitalized patients

**DOI:** 10.1042/BSR20222362

**Published:** 2023-03-16

**Authors:** Nosayba Al-Azzam, Basheer Khassawneh, Sayer Al-Azzam, Reema A. Karasneh, Mamoon A. Aldeyab

**Affiliations:** 1 Department of Physiology and Biochemistry, Faculty of Medicine, Jordan University of Science and Technology, Irbid, Jordan; 2 Department of Internal Medicine, Faculty of Medicine, Jordan University of Science and Technology, Irbid, Jordan; 3 Department of Clinical Pharmacy, Faculty of Pharmacy, Jordan University of Science and Technology, Irbid, Jordan; 4 Department of Basic Medical Sciences, Faculty of Medicine, Yarmouk University, Irbid, Jordan; 5 Department of Pharmacy, School of Applied Sciences, University of Huddersfield, Huddersfield, U.K.

**Keywords:** Acid-base balance, Arterial blood gas, COVID-19, Mortality, Respiratory and metabolic acidosis, Respiratory and metabolic alkalosis

## Abstract

Severe coronavirus disease 2019 (COVID-19) infection can lead to extensive lung infiltrate, a significant increase in the respiratory rate, and respiratory failure, which can affect the acid–base balance. No research in the Middle East has previously examined acid–base imbalance in COVID-19 patients. The present study aimed to describe the acid–base imbalance in hospitalized COVID-19 patients, determine its causes, and assess its impact on mortality in a Jordanian hospital.

The study divided patients into 11 groups based on arterial blood gas data. Patients in normal group were defined as having a pH of 7.35–7.45, PaCO_2_ of 35–45 mmHg, and HCO_3_^−^ of 21–27 mEq/L. Other patients were divided into 10 additional groups: mixed acidosis and alkalosis, respiratory and metabolic acidosis with or without compensation, and respiratory and metabolic alkalosis with or without compensation. This is the first study to categorize patients in this way.

The results showed that acid–base imbalance was a significant risk factor for mortality (*P*<0.0001). Mixed acidosis nearly quadruples the risk of death when compared with those with normal levels (OR = 3.61, *P*=0.05). Furthermore, the risk of death was twice as high (OR = 2) for metabolic acidosis with respiratory compensation (*P*=0.002), respiratory alkalosis with metabolic compensation (*P*=0.002), or respiratory acidosis with no compensation (*P*=0.002).

In conclusion, acid–base abnormalities, particularly mixed metabolic and respiratory acidosis, were associated with increased mortality in hospitalized COVID-19 patients. Clinicians should be aware of the significance of these abnormalities and address their underlying causes.

## Introduction

The new strain of the Severe Acute Respiratory Syndrome Coronavirus 2 (SARS-CoV-2) disease, which is known as COVID-19, spreads through sneezing and coughing droplets [1]. Coronavirus disease 2019 (COVID-19) spread worldwide after emerging in Wuhan, China. COVID-19 affects many systems of the human being, mainly the respiratory system, through acting on angiotensin-converting enzyme 2 (ACE-2) receptors that are located at the surface of respiratory cells [2,3]. COVID-19 has a huge impact on other systems that harbor ACE-2 receptors on their surfaces, such as the cardiovascular, neurological, gastrointestinal, and musculoskeletal systems [4]. SARS-CoV-2 is a highly contagious virus [5], and infected individuals are infectious before symptoms appear [4]. However, estimates differ on the significance of asymptomatic people spreading the virus [6]. SARS-CoV-2 can cause a variety of symptoms, including asymptomatic infection and severe pneumonia-induced death [5].

The clinical range of the disease manifests as mild, moderate, or severe illness [7,8]. Intensive therapy, which includes non-invasive and invasive ventilation, antipyretics, antivirals, antibiotics, and steroids, is necessary for moderate and severe cases, which also call for hospitalization. Plasma exchange therapy and immunomodulatory drugs may be necessary for the treatment of complicated cases [7]. A COVID-19 infection can lead to a deterioration of lung capacity brought on by pulmonary interstitial fibrosis [9]. SARS-CoV-2, on the other hand, causes excessive and prolonged cytokine and chemokine responses in some infected individuals, a phenomenon known as the ‘cytokine storm’. Cytokine storms cause acute respiratory distress syndrome (ARDS), or multiple-organ dysfunction, which leads to physiological deterioration and death [10].

Several predictors of COVID-19 severity and mortality have been identified [11]. In a retrospective, observational cohort study of 3988 consecutive critically ill patients with laboratory-confirmed COVID-19 referred for ICU admission to the coordinating center in Italy, it was discovered that independent risk factors associated with mortality included older age, male sex, and histories of chronic obstructive pulmonary disease, hypercholesterolemia, and Type 2 diabetes mellitus [12]. Another study conducted in Jordan confirmed that several risk factors, such as older age, smoking, admission severity status, comorbidities, and lab test results, were linked to COVID-19 mortality [13].

A normal acid–base balance is necessary to ensure appropriate physiology and cell activity. The occurrence of any acid–base abnormalities increases the probability of experiencing a negative consequence [14,15]. Acid–base disorders are classified as either respiratory or metabolic, depending on the carbon dioxide (CO_2_) tension and the bicarbonate ions (HCO_3_^−^) levels in physiological fluids [16,17]. Acid–base changes are caused by a number of illnesses, including respiratory failure, shock, renal failure, and hepatic failure [18]. An early and correct identification of an acid–base imbalance is required to improve the outcome because severe acid–base derangements can be life-threatening [15].

As a common acid–base disorder in COVID-19 patients, respiratory alkalosis was linked to an increased risk of severe events [19]. Understanding normal physiological function is the first step in interpreting acid–base disorders. To maintain acid–base balance, the body employs buffering processes, ventilation rate, and renal mechanisms. The use of pH, pCO_2_, and HCO_3_^−^ as coordinates can help to identify compensation and mixed acid–base disorders [17,20]. In a retrospective study of 112 COVID-19 patients who were hospitalized at the University Hospital of Modena, it was discovered that 79.7% of the patients had abnormal acid–base balances. Metabolic alkalosis was the most significant change, and it was followed by respiratory alkalosis, combined alkalosis, respiratory acidosis, metabolic acidosis, and other compensated acid–base disturbances, in that order [21]. Another study that included 105 COVID-19 patients with ARDS within the first 48 h of needing noninvasive respiratory support discovered that the majority of the patients had respiratory alkalosis. Metabolic alkalosis, the second more common acid–base disorder, was mentioned. Only a small percentage of the patients had respiratory acidosis, and none of the patients had metabolic acidosis [22].

Although there have been some studies about acid–base imbalance in COVID-19 patients, there have been none from the Middle East. Consequently, the present study’s objectives were to describe the acid–base imbalance in hospitalized COVID-19 patients, identify the risk factors for this imbalance, and assess how it affected in-hospital mortality for COVID-19 patients in Jordan. In addition, the present study aimed to describe the clinical characteristics of patients with and without acid–base imbalances and examine the association between distinct acid–base imbalances and the outcome of adult COVID-19 patients.

## Methodology

### Data sources and research design

This retrospective study was carried out at King Abdullah University Hospital (KAUH), a tertiary hospital and one of the largest medical structures in Jordan. Patients with COVID-19 who were admitted to KAUH between September 20, 2020, and August 8, 2021, whose arterial blood gas (ABG) measurements upon admission were reported, and whose SARS-CoV-2 nasopharyngeal swab polymerase chain reaction (PCR) confirmed positivity, were included in the study.

Patients under the age of 18 and those with missing ABG parameters on admission or who were asymptomatic were excluded from the study. KAUH is a university teaching hospital, so all data are consented to be used in cohort studies once the patient agreed to be treated in the hospital and the IRB committee approved the study.

### Research variables

Electronic hospital records were used to identify patients’ clinical data, such as vital signs, comorbidities, and hospitalization course and outcomes. Laboratory results were also included in the study alongside age, gender, smoking status, height, and weight. The body mass index (BMI) was computed using the formula BMI = weight (kg)/height^2^ (m^2^) and categorized according to World Health Organization (WHO) guidelines [23]. Comorbidities were identified using related International Classification of Diseases (ICD) codes, and laboratory results at admission were interpreted using hospital laboratory reference values. According to the National Institutes of Health’s (NIH) Clinical Spectrum of SARS-CoV-2 Infection [24], the severity of the patient’s condition upon admission was classified. Patients with a positive test but no symptoms were classified as ‘asymptomatic’, and those exhibiting multiple symptoms but no respiratory distress were classified as having ‘mild illness’. Patients with lower respiratory disease on clinical assessment or imaging and an oxygen saturation (SpO_2_) of 94% or more on room air at sea level were classified as having ‘moderate illness’. Patients were classified as having ‘severe illness’ when they exhibited characteristics such as a SpO_2_ of less than 94%, a PaO2/FiO2 of less than 300 mmHg, a respiratory rate of more than 30 breaths per minute, or lung infiltrates of more than 50%. Patients with respiratory failure, septic shock, and/or multiple organ dysfunction were considered ‘critically ill’.

## Patient classification

ABG values at admission were used to categorize patients. Table 1 displays the ranges used to categorize the patients. Patients were deemed normal only if their laboratory arterial blood pH was between 7.35 and 7.45, PaCO_2_ was between 35 and 45 mmHg, and HCO_3_^−^ level was between 21 and 27 mEq/L. Patients with respiratory acidosis had a high CO_2_ concentration (>45) and a pH less than 7.4, whereas patients with respiratory alkalosis had a low CO_2_ concentration (<35) and a pH greater than 7.4. On the other hand, metabolic alkalosis is considered when HCO_3_^−^ exceeds 27 mEq/L and pH exceeds 7.4, whereas metabolic acidosis occurs when pH falls below 7.4 and HCO_3_^−^ falls below 21 mEq/L. The compensatory mechanisms from the respiratory or metabolic sides were also considered in categorizing the patients, as illustrated in Table 1.

**Table 1:** Reference values for patients’ classification

Patients’ classification	pH	CO_2_	HCO_3_^−^
Normal	7.35–7.45	35–45	21–27
Mixed acidosis	<7.35	>45	<21
Mixed alkalosis	>7.45	<35	>27
Respiratory acidosis with compensation (RAC)	<7.4	>45	>27
Respiratory acidosis with no compensation (RANC)	<7.4	>45	21–27
Respiratory alkalosis with compensation (RAlkC)	>7.4	<35	<21
Respiratory alkalosis with no compensation (RAlkNC)	>7.4	<35	21–27
Metabolic acidosis with compensation (MAC)	<7.4	<35	<21
Metabolic acidosis with no compensation (MANC)	<7.4	35-45	<21
Metabolic alkalosis with compensation (MAlkC)	>7.4	>45	>27
Metabolic alkalosis with no compensation (MAlkNC)	>7.4	35-45	>27

### Statistical analysis

The analysis began with categorizing the patients based on their ABG values on admission. Then, a distributional study of patient features was done across all acid–base classification groups. Summary tables were generated to investigate the proportion of COVID-19 inpatients by age group, gender, and clinical features within each acid–base group. To investigate statistical differences in the frequencies of the categorical groupings, the acid–base status linked with each attribute was studied using χ^2^ tests. Furthermore, mortality rates, mechanical breathing requirements, and disease severity were assessed using χ^2^ tests to assess statistical differences across acid–base-categorized groups. In addition to ratio testing, we utilized a nominal logistic regression model to calculate the odds ratio and statistical significance for each of the investigated components and the acid-base status. A two-sided *P* ≤ 0.05 was regarded as statistically significant.

## Results

### Demographic data of the enrolled patients

A total of 1233 patients admitted to KAUH with confirmed COVID-19 met the inclusion criteria and were included in the present study during the study period. Based on their arterial blood pH, PaCO_2_, and HCO_3_^−^ levels, we classified the patients into 11 groups.

Table 2 shows the patients’ blood acid–base state based on their characteristics, comorbidities, and laboratory potassium test. A total of 613 patients (49.7%) were above the age of 65, 718 (58.2%) were men, 527 (42.7%) were obese, 781 (63.3%) had hypertension, and 654 (53.0%) were diabetic.

**Table 2:** Patient acid-base classification versus demographic variables, comorbidities, and potassium levels

Count row%	Total (column%)	Normal	Mixed acidosis	Mixed alkalosis	RAC	RANC	RAlkC	RAlkNC	MAC	MANC	MAlkC	MAlkNC	*P*-value
**Age**													
18–40	100 (8.1)	25 (25.0)	0 (0.0)	0 (0.0)	7 (7.0)	10 (10.0)	18 (18.0)	17 (17.0)	13 (13.0)	4 (4.0)	3 (3.0)	3 (3.0)	0.150
41–65	520 (42.2)	141 (27.1)	8 (1.5)	3 (0.6)	30 (5.8)	52 (10.0)	77 (14.8)	105 (20.2)	46 (8.9)	26 (5.0)	13 (2.5)	19 (3.7)	
>65	613 (49.7)	169 (27.6)	10 (1.6)	0 (0.0)	36 (5.9)	50 (8.2)	77 (12.6)	91 (14.9)	81 (13.2)	51 (8.3)	20 (3.3)	28 (4.6)	
**Gender**													
Male	718 (58.2)	201 (28.0)	8 (1.1)	1 (0.1)	41 (5.7)	61 (8.5)	105 (14.6)	130 (18.1)	77 (10.7)	43 (6.0)	23 (3.2)	28 (3.9)	0.754
Female	515 (41.8)	134 (26.0)	10 (1.9)	2 (0.4)	32 (6.2)	51 (9.9)	67 (13.0)	83 (16.1)	63 (12.2)	38 (7.4)	13 (2.5)	22 (4.3)	
**BMI**													
Underweight	2 (0.2)	0 (0.0)	0 (0.0)	0 (0.0)	0 (0.0)	0 (0.0)	0 (0.0)	1 (50.0)	1 (50.0)	0 (0.0)	0 (0.0)	0 (0.0)	0.723
Normal	175 (14.2)	39 (22.3)	4 (2.3)	0 (0.0)	8 (4.6)	22 (12.6)	29 (16.6)	24 (13.7)	27 (15.4)	11 (6.3)	7 (4.0)	4 (2.3)	
Obese	527 (42.7)	141 (26.8)	5 (1.0)	1 (0.2)	37 (7.0)	48 (9.1)	72 (13.7)	93 (17.7)	52 (9.9)	34 (6.5)	18 (3.4)	26 (4.9)	
Overweight	418 (33.9)	121 (29.0)	8 (1.9)	2 (0.5)	19 (4.5)	34 (8.1)	59 (14.1)	71 (17.0)	52 (12.4)	28 (6.7)	9 (2.2)	15 (3.6)	
Missing	111 (9.0)	34 (30.6)	1 (0.9)	0 (0.0)	9 (8.1)	8 (7.2)	12 (10.8)	24 (21.6)	8 (7.2)	8 (7.2)	2 (1.8)	5 (4.5)	
**Smoking status**													
Non-smoker	908 (73.6)	259 (28.5)	14 (1.5)	2 (0.2)	53 (5.8)	79 (8.7)	119 (13.1)	151 (16.6)	99 (10.9)	64 (7.1)	29 (3.2)	39 (4.3)	0.505
Ex-smoker	157 (12.7)	44 (28.0)	1 (0.6)	0 (0.0)	7 (4.5)	16 (10.2)	26 (16.6)	24 (15.3)	18 (11.5)	10 (6.4)	4 (2.6)	7 (4.5)	
Active smoker	168 (13.6)	32 (19.0)	3 (1.8)	1 (0.6)	13 (7.7)	17 (10.1)	27 (16.1)	38 (22.6)	23 (13.7)	7 (4.2)	3 (1.8)	4 (2.4)	
**Comorbidities**													
HTN	781 (63.3)	207 (26.5)	17 (2.2)	3 (0.4)	49 (6.3)	70 (9.0)	99 (12.7)	124 (15.9)	101 (12.9)	63 (8.1)	21 (2.7)	27 (3.5)	**0.001**
DM	654 (53.0)	169 (25.8)	14 (2.1)	3 (0.5)	39 (6.0)	60 (9.2)	78 (11.9)	93 (14.2)	96 (14.7)	59 (9.0)	21 (3.2)	22 (3.4)	**0.0001**
Dyslipidemia	69 (5.6)	16 (23.2)	5 (7.2)	0 (0.0)	4 (5.8)	5 (7.3)	8 (11.6)	12 (17.4)	6 (8.7)	9 (13.0)	3 (4.3)	1 (1.4)	**0.005**
IHD	243 (19.7)	69 (28.4)	6 (2.5)	0 (0.0)	13 (5.4)	20 (8.2)	33 (13.6)	38 (15.6)	34 (14.0)	16 (6.6)	5 (2.1)	9 (3.7)	0.759
Atrial fibrillation	53 (4.3)	15 (28.3)	2 (3.8)	0 (0.0)	2 (3.8)	6 (11.3)	7 (13.2)	9 (17.0)	6 (11.3)	1 (1.9)	4 (7.6)	1 (1.9)	0.485
Heart failure	121 (9.8)	30 (24.8)	3 (2.5)	1 (0.8)	11 (9.1)	10 (8.3)	15 (12.4)	15 (12.4)	12 (9.9)	13 (10.7)	4 (3.3)	7 (5.8)	0.257
Asthma	44 (3.6)	9 (20.4)	0 (0.0)	0 (0.0)	5 (11.4)	9 (20.5)	6 (13.7)	5 (11.4)	2 (4.5)	1 (2.3)	2 (4.6)	5 (11.4)	**0.023**
COPD	15 (1.2)	3 (20.0)	0 (0.0)	0 (0.0)	1 (6.7)	5 (33.3)	1 (6.7)	0 (0.0)	2 (13.3)	0 (0.0)	0 (0.0)	3 (20.0)	**0.006**
Chronic kidney disease	118 (9.6)	20 (16.9)	5 (4.2)	0 (0.0)	7 (5.9)	9 (7.6)	12 (10.2)	6 (5.1)	35 (29.7)	19 (16.1)	3 (2.5)	2 (1.7)	**<.0001**
ESRD	32 (2.6)	6 (18.8)	1 (3.1)	0 (0.0)	1 (3.1)	5 (15.6)	2 (6.2)	1 (3.1)	12 (37.5)	3 (9.4)	0 (0.0)	1 (3.1)	**0.001**
Immunocompromised	52 (4.2)	11 (21.1)	2 (3.9)	1 (1.9)	3 (5.8)	3 (5.8)	6 (11.5)	10 (19.2)	9 (17.3)	4 (7.7)	2 (3.8)	1 (1.9)	0.240
Malignancy	96 (7.8)	24 (25.0)	3 (3.1)	1 (1.0)	3 (3.1)	8 (8.3)	6 (6.3)	20 (20.8)	11 (11.5)	11 (11.5)	3 (3.1)	6 (6.3)	0.085
**Potassium laboratory test**													
Low	105 (8.5)	12 (11.4)	1 (0.95	1 (0.95)	6 (5.7)	6 (5.7)	14 (13.3)	28 (26.7)	8 (7.6)	5 (4.8)	7 (6.7)	17 (16.2)	**0.0001**
Normal	879 (71.3)	261 (29.7)	12 (1.4)	2 (0.2)	50 (5.7)	75 (8.5)	131 (14.9)	154 (17.5)	94 (10.7)	49 (5.6)	25 (2.8)	26 (3.0)	
High	97 (7.9)	21 (21.7)	5 (5.2)	0 (0.0)	5 (5.2)	14 (14.4)	6 (6.2)	5 (5.2)	20 (20.6)	19 (19.6)	1 (1.0)	1 (1.0)	
Missing	152 (12.3)	41 (27.0)	0 (0.0)	0 (0.0)	12 (7.9)	17 (11.2)	21 (13.8)	26 (17.1)	18 (11.8)	8 (5.3)	3 (2.0)	6 (4.0)	
Total (%)	1,233	335 (27.2)	18 (1.5)	3 (0.2)	73 (5.9)	112 (9.1)	172 (14.0)	213 (17.3)	140 (11.4)	81 (6.6)	36 (2.9)	50 (4.1)	

**Figure 1:**
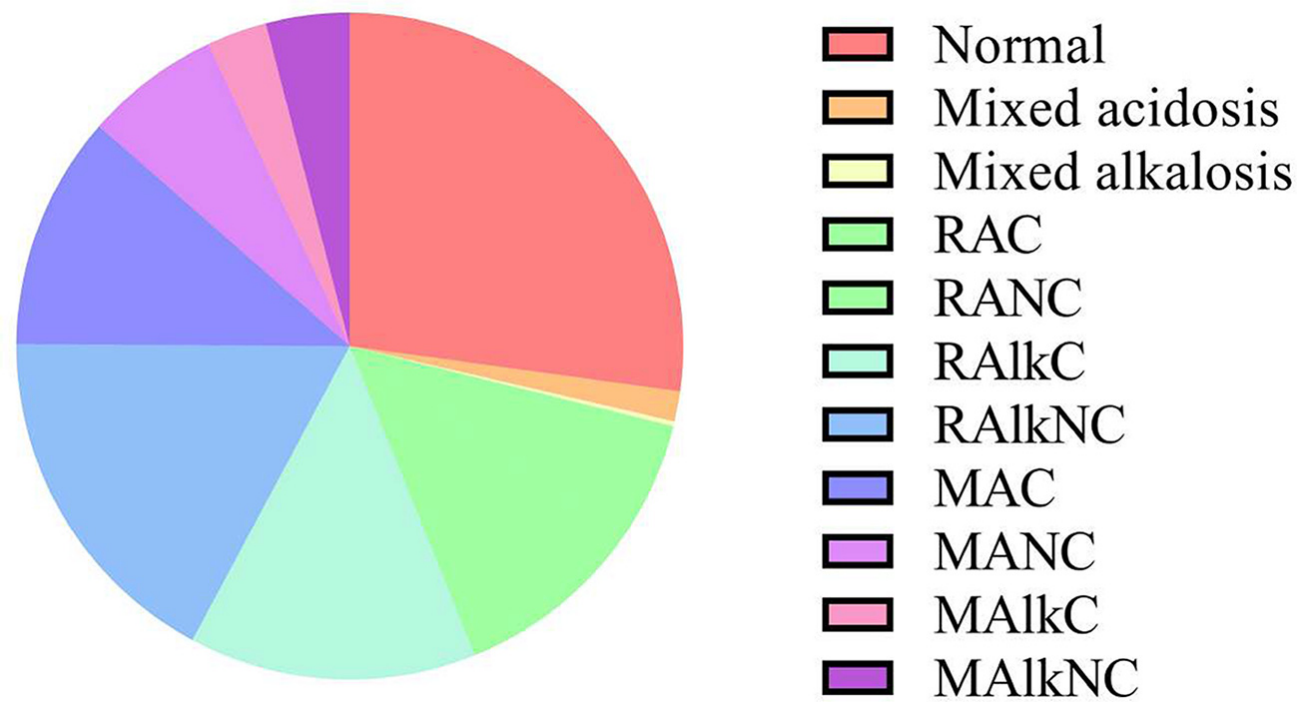
The distribution of acid–base status among patients hospitalized due to COVID-19 The pie chart represents the distribution of the hospitalized COVID-19 patients according to their acid–base status. Most patients (27.2%) had normal ABG levels. The distribution of patients with abnormal ABG values was as follows: mixed acidosis (1.5%), mixed alkalosis (0.2%), RAC (5.9%), RANC (9.1%), RAlkC (14.0%), RAlkNC (17.3%), MAC (11.4%), MANC (6.6%), MAlkC (2.9%), and MAlkNC (4.1%).

The distribution of acid–base status among patients hospitalized due to COVID-19 is shown in Figure 1. Among all acid–base classified groups, the highest percent (27.2%) was for patients who had normal ranges for all three measures, followed by respiratory alkalosis with no compensation (RAlkNC) (17.3%), respiratory alkalosis with compensation (RAlkC) (14.0%), and metabolic acidosis with compensation (MAC) (11.4%).

Normal-parameter patients were predominantly elderly (>65 years old, *n*=169), males (*n*=201), obese (*n*=141), and nonsmokers (*n*=259). Most patients among all different variable groups (highest percent in a row), according to the parameters analyzed, are shown to have normal acid–base values, with the exception of those who were active smokers, had asthma, chronic obstructive pulmonary disease (COPD), chronic kidney disease (CKD), end-stage renal disease (ESRD), or have low potassium levels. The bulk of the patients who were underweight were not in the normal category either.

As the numbers show in Table 2, RAlkNC was found to have the highest percent of acid–base imbalance among active smokers (22.6%). Among asthmatics, the dominant classification was respiratory acidosis with no compensation (RANC) (20.5%) or normal (20.4%) acid–base levels, whereas the main acid–base imbalance among COPD patients was RANC (33.3%). While MAC was the major acid–base classification among patients with chronic renal disease (29.7%) and ESRD (37.5%).

Further, hypertension, diabetes, dyslipidemia, asthma, COPD, CKD, and ESRD comorbidities, as well as potassium levels, were the only factors found to have a significant impact on acid–base status (Table 2). Among the laboratory findings (Supplementary Table S1) from COVID-19 patients, only the potassium test was a significant factor, as shown in Table 2. The majority of patients have normal kidney function test (KFT) potassium testing (*n*=879). The highest percent of patients with low potassium levels had RAlkNC (26.7%) and RAlkC (13.3%). Not surprisingly, the highest percentages of patients with normal potassium KFT also have normal ABG values. In descending order, the highest precents of patients with increased potassium levels had normal ABG levels (21.7%), MAC (20.6%), and metabolic acidosis with no compensation (MANC) (19.6%).

Then, we investigated the impact of acid–base status on illness severity. The severity of the patients is shown in Table 3 by their acid–base status. The severity of the condition has no significant bearing on the outcome (acid–base balance) (*P*=0.3913). The majority of patients (57% of total) were classified as critical cases, while the minority (1.1% of total) were classified as mild. Critical cases were found in 62.9% of RAlkNC, 61.1% of mixed acidosis as well as RAlkC, 60.3% of respiratory acidosis with compensation (RAC), 58.9% of RANC, and 58.3% of metabolic alkalosis with compensation (MAlkC) cases.

**Table 3:** Effect of acid–base status on disease severity, the need for mechanical ventilator, and survival

Count row (%)	Total	Severity				*P* value	Invasive mechanical ventilators need		*P* value	Survival		*P* value
		Mild	Moderate	Severe	Critical		No	Yes		Survived	Deceased	
**Normal**	335 (27.2)	4 (1.2)	40 (11.9)	106 (31.6)	185 (55.2)	0.3913	259 (77.3)	76 (22.7)	<0.0001	215 (64.2)	120 (35.8)	<0.0001
**Mixed acidosis**	18 (1.5)	0 (0.0)	3 (16.7)	4 (22.2)	11 (61.1)		3 (16.7)	15 (83.3)		4 (22.2)	14 (77.8)	
**Mixed alkalosis**	3 (0.24)	0 (0.0)	0 (0.0)	3 (100)	0 (0.0)		2 (66.7)	1 (33.3)		1 (33.3)	2 (66.7)	
**RAC**	73 (5.9)	0 (0.0)	9 (12.3)	20 (27.4)	44 (60.3)		52 (71.2)	21 (28.8)		44 (60.3)	29 (39.7)	
**RANC**	112 (9.1)	3 (2.7)	19 (17.0)	24 (21.4)	66 (58.9)		82 (73.2)	30 (26.8)		59 (52.7)	53 (47.3)	
**RAlkC**	172 (14.0)	0 (0.0)	16 (9.3)	51 (29.7)	105 (61.1)		117 (68.0)	55 (32.0)		80 (46.5)	92 (53.5)	
**RAlkNC**	213 (17.3)	3 (1.4)	20 (9.4)	56 (26.3)	134 (62.9)		164 (77.0)	49 (23.0)		124 (58.2)	89 (41.8)	
**MAC**	140 (11.4)	2 (1.4)	22 (15.7)	42 (30.0)	74 (52.9)		95 (67.9)	45 (32.1)		56 (40.0)	84 (60.0)	
**MANC**	81 (6.6)	2 (2.5)	12 (14.8)	27 (33.3)	40 (49.4)		55 (67.9)	26 (32.1)		38 (46.9)	43 (53.1)	
**MAlkC**	36 (2.9)	0 (0.0)	5 (13.9)	10 (27.8)	21 (58.3)		27 (75.0)	9 (25.0)		19 (52.8)	17 (47.2)	
**MAlkNC**	50 (4.1)	0 (0.0)	4 (8.0)	18 (36.0)	28 (56.0)		37 (74.0)	13 (26.0)		33 (66.0)	17 (34.0)	
**Total**	1233	14 (1.1)	150 (12.2)	361 (29.3)	708 (57.4)		893 (72.4)	340 (27.6)		673 (54.6)	560 (45.4)	

We also investigated the requirement for an invasive mechanical ventilator (Table 3). In total, 340 patients (27.6%) required invasive mechanical ventilation. The demand for an invasive mechanical ventilator is substantially influenced by acid–base status (*P*<000.1). Among the acid–base categories, the normal group had the lowest percentage of those who needed mechanical ventilation. Mixed acidosis was the only category that had a larger proportion of patients who needed mechanical ventilation (83.3%) than those who did not.

Then, we explored the impact of the patients’ acid–base status on their survival (Table 3). About 54.6% of all enrolled patients survived, while 45.4% perished. The acid–base state has a substantial effect on survival (*P*<0.0001). The metabolic alkalosis with no compensation (MAlkNC) (66.0%) and normal (64.2%) groups had the highest survival percentages among the acid–base classed groups. Among the acid–base classed groups, MAC (60%), MANC (53.1%), mixed acidosis (77.8%), mixed alkalosis (66.7%), RAlkC (53.5%), and MANC (53.1%) were more common in deceased patients than in survivors.

Since demographics, comorbidities, and laboratory tests influenced acid-base status and could be a risk factor for mortality, the effect of acid–base status on the death could be attributable to the other investigated factors. To confirm the link between acid–base status and mortality, a regression model that included all analyzed covariates (demographics, comorbidities, and laboratory tests) along with acid–base status was utilized. As numbers show in Table 4, being an older patient (OR = 2.16, CI = 1.248–3.728), an active smoker (OR = 1.8, CI = 1.215–2.667), or having CKD (OR = 1.99, CI = 1.243–3.195) all raise the likelihood of death. In addition, having high levels of sodium, C-reactive protein, AST, or low levels of albumin also raise the risk of death (OR > 1, *P*<0.05).

**Table 4:** Regression model of death versus survival

Level	Odds ratio	*P*-value	Lower 95%	Upper 95%
**Age** (18–40 age group is a reference)				
41–65	1.25	0.414	0.733	2.129
>65	2.16	**0.006**	1.248	3.728
**Smoking status** (Non-smoker is a reference)				
Active smoker	1.80	**0.003**	1.215	2.667
Ex-smoker	1.30	0.210	0.864	1.950
**CKD** (Yes vs. no)	1.99	**0.004**	1.243	3.195
**CRP** (High vs. low)	4.31	**0.031**	1.142	16.292
**Sodium** (High vs. Normal)	7.75	**<0.0001**	2.911	20.616
**Albumin (Low vs. Normal)**	1.68	**0.0003**	1.267	2.2404
**AST (High vs. Normal)**	2.17	**<0.0001**	1.589	2.9660
**Acid-base status** (Normal is a reference)				
MAC	2.12	**0.002**	1.332	3.359
MAlkC	1.42	0.370	0.660	3.056
MAlkNC	0.66	0.258	0.318	1.359
MANC	1.42	0.222	0.809	2.492
Mixed acidosis	3.61	**0.049**	1.004	12.975
Mixed alkalosis	3.06	0.390	0.239	39.157
RAC	1.00	0.999	0.560	1.786
RAlkC	1.94	**0.002**	1.283	2.925
RAlkNC	1.18	0.404	0.796	1.760
RANC	1.76	**0.022**	1.087	2.862

Abbreviations: MAC, metabolic acidosis with compensation; MAlkC, metabolic alkalosis with compensation; MAlkNC, metabolic alkalosis with no compensation; MANC, metabolic acidosis with no compensation; RAC, respiratory acidosis with compensation; RAlkC, respiratory alkalosis with compensation; RAlkNC, respiratory alkalosis with no compensation; RANC, respiratory acidosis with no compensation.

Even so, the acid–base status remains a substantial risk factor. When compared with those with normal levels, mixed acidosis increases the fatality risk almost four times (OR = 3.61, *P*=0.049). Furthermore, MAC, RAlkC, or RANC doubles the mortality risk when compared with normal.

Finally, a nominal logistic regression analysis was performed to determine factors associated with the occurrence of acid–base imbalance (Table 5). Variables like current smoking (OR: 1.85, *P*=0.007), CKD (OR: 2.01, *P*=0.012), high LDH (OR: 2.55, *P*=0.0425), low potassium (OR: 4.27, *P*<0.0001), and high AST (OR: 1.72, *P*=0.0017) were associated with higher odds of acquiring an acid–base imbalance.

**Table 5:** Nominal logistic regression analysis for factors associated with the occurrence of acid–base imbalance

Variable	OR	*P*-value	Lower 95%	Upper 95%
Current smoker	1.85	**0.007**	1.182	2.881
CKD	2.01	**0.012**	1.166	3.457
High LDH	2.55	**0.0425**	1.032	6.318
Low potassium	4.27	**<.0001**	2.191	8.307
High AST	1.72	**0.0017**	1.226	2.408

Abbreviations: AST, aspartate aminotransferase; CKD, chronic kidney disease; LDH, lactate dehydrogenase.

## Discussion

The present study is the first comprehensive report of the range of acid–base changes as measured by ABG levels among hospitalized COVID-19 patients at a Jordanian hospital. COVID-19 is a highly contagious virus that causes pneumonia [25], and pneumonia is one of the leading causes of respiratory alkalosis [17]. As a result, we decided to investigate acid–base imbalance in COVID-19 patients. Surprisingly, the group that had the highest number of hospitalized patients was the normal group (*n*=335, 27.2%). A separate study discovered that just 25% of COVID-19 patients admitted to the ICU had normal acid–base balance, whereas 61% had alkalosis. However, the categorization process used in our investigation and the previously cited study differed [26]. The most common acid–base abnormalities were RAlkNC (17.3%), RAlkC (14.0%), and MAC (11.4%). Similarly, respiratory alkalosis was reported to be the most common acid–base disorder in patients hospitalized due to COVID-19 infection, accounting for 40% of the study population [22]. This study found that the rate of respiratory alkalosis is approximately 2-fold higher than respiratory acidosis (31.3% vs. 15% of the study populations). Similar findings were observed in two previous studies [21,22]. One study found that the occurrence of respiratory alkalosis in COVID-19 patients is due to hyperventilation induced by hypoxia (PO_2_ = 70.1 ± 32.9 mmHg). Moreover, patients with respiratory failure with hypercapnia (type 2 respiratory failure) were found to experience respiratory acidosis (PCO_2_ = 62.2 ± 13.4 mmHg) [21].

In the present study, hypertension, diabetes mellitus, dyslipidemia, asthma, COPD, CKD, and ESRD comorbidities, as well as potassium levels, were shown in the results to have a significant impact on acid-base status. In a study by Alfano et al., it was demonstrated that the presence of CKD and the levels of potassium have a significant effect on the acid-base status of hospitalized COVID-19 patients, similar to our findings [21]. In the present study, the majority of diabetic individuals had normal ABG readings, and metabolic acidosis with compensation coming in second. This can be understood since diabetes is a common cause of ketoacidosis, which is a kind of metabolic acidosis [27]. Asthma and COPD can both produce hypoventilation and, as a result, respiratory acidosis [28,29]. In line with this, the highest percent of asthmatic patients in our study exhibited uncompensated respiratory acidosis.

The kidneys are responsible for maintaining the body’s acid–base balance by reabsorbing bicarbonate that has been filtered by the glomeruli and excreting titratable acids and ammonia through the urine. Acid retention and metabolic acidosis occur in CKD when renal function declines [30]. The highest percent of CKD patients recruited in the present study were additionally compensated for metabolic acidosis. Patients with ESRD undergoing hemodialysis are at risk of interdialytic acid buildup and chronic acidosis-related consequences, including mortality [31]. In line with this, the majority of ESRD patients who participated in this trial had MAC.

Despite the fact that only 18 patients had mixed acidosis, 14 of those patients died. Furthermore, when compared with normal, mixed acidosis quadruples the fatality risk. The most frequently reported acid–base derangement in non-COVID-19 critically ill patients is severe mixed acidemia. This was observed in 6% of critically ill patients and was associated with a 57% ICU mortality rate [32]. Our results showed that 83.3% of mixed acidosis patients needed mechanical ventilation during their hospital stay, and 77.8% of them passed away.

RAlkC is found in 14.0% of patients and has been shown to be a key factor in mortality. When compared with normal patients, it doubled the death risk. In a Chinese study of 230 adult COVID-19 patients, 66 patients (28.7%) showed respiratory alkalosis (pH > 7.45, PaCO_2_ < 35 mmHg) on admission, and those patients have an increased chance of developing severe cases [19]. In our study, 31% exhibited respiratory alkalosis, with 14.0% compensated and 17.3% uncompensated instances. Although severity was not a statistically significant factor in our study, approximately 60% of respiratory acidosis cases were critical. Another study found that 24 out of 32 (75%) individuals with CO_2_ levels <35 mm Hg died [33]. However, this percent may include cases of pulmonary alkalosis and mixed alkalosis.

When compared to patients with normal ABG readings, RANC and MAC doubled the risk of fatality. Lung-protective ventilation in COVID-19 respiratory failure may result in severe respiratory acidosis without considerable hypoxemia [34]. The higher mortality rate in patients with respiratory acidosis may be due to the binding of protein S to angiotensin-converting enzyme 2 receptors, which penetrate alveolar epithelial cells, resulting in direct toxic effects and an overactive immune response. This causes a systemic inflammatory response, resulting in a cytokine storm and lung tissue harm. Acute respiratory distress syndrome and metabolic acidosis can occur in severe cases [35]. Approximately 52.9% of MAC cases in our study were critical cases, and 60% of them deceased.

The significant association between the presence of acid–base derangements and increased mortality among hospitalized patients with severe COVID-19 infection should alert treating clinicians to the importance of addressing these abnormalities. Understanding the underlying etiology for acid–base disorders and early intervention for metabolic and respiratory acidosis may help to improve the outcome of these patients. Moreover, early identification of major acid–base disorders like mixed respiratory and metabolic acidosis may help to identify patients with poor prognoses.

The present study has a few limitations. It utilized inpatient record data from a single tertiary hospital in the north of Jordan, and data collection was retrospective. In addition, our study was limited to the acid–base status at the time of admission. Future prospective research on the dynamic acid–base status may be required.

## Conclusion

Acid–base imbalance in hospitalized patients with COVID-19 is a risk factor for mortality, especially mixed respiratory and metabolic acidosis. Rigorous acid–base monitoring during COVID-19 hospitalization should be required in order to identify patients at increased risk of death. Early detection of such derangements may help to prevent future clinical derangements and hence improve survival.

## Supplementary Material

Supplementary Table S1

## Data Availability

Authors agree to make any materials, data, code, and associated protocols available upon request.

## Abbreviations

AST: aspartate aminotransferase
CKD: chronic kidney disease
LDH: lactate dehydrogenase
MAC: metabolic acidosis with compensation
MAlkC: metabolic alkalosis with compensation
MAlkNC: metabolic alkalosis with no compensation
MANC: metabolic acidosis with no compensation
RAC: respiratory acidosis with compensation
RAlkC: respiratory alkalosis with compensation
RAlkNC: respiratory alkalosis with no compensation
RANC: respiratory acidosis with no compensation
SARS-CoV-2: Severe Acute Respiratory Syndrome Coronavirus 2
