# Supplementary material for: Acid–base imbalance as a risk factor for mortality among COVID-19 hospitalized patients
Source: Biosci Rep. 2023 Mar 16;43(3):BSR20222362. doi: 10.1042/BSR20222362 (PMC10037419; doi:10.1042/BSR20222362)
Supplement: Supplementary Table S1 [file BSR-2022-2362_supp.pdf]

Supplementary Table 1: Patient acid-base classification vs. laboratory tests.

Count<br>Row %	Total	Normal	Mixed acid	Mixed alkal	RAC	RANC	RAlkC	RAlkNC	MAC	MANC	MAlkC	MAlkNC	P value
**C-Reactive Protein**													
Low	22<br>(1.8)	7<br>(31.8)	0<br>(0.0)	0<br>(0.0)	3<br>(13.6)	4<br>(18.2)	2<br>(9.1)	2<br>(9.1)	1<br>(4.6)	0<br>(0.0)	0<br>(0.0)	3<br>(13.6)	0.2653
High	886<br>(71.9)	247<br>(27.9)	12<br>(1.4)	1<br>(0.1)	52<br>(5.9)	74<br>(8.4)	123<br>(13.9)	163<br>(18.4)	100<br>(11.3)	55<br>(6.2)	23<br>(2.6)	36<br>(4.1)	
Missing	325<br>(26.4)	81<br>(24.9)	6<br>(1.9)	2<br>(0.6)	18<br>(5.5)	34<br>(10.5)	47<br>(14.5)	48<br>(14.8)	39<br>(12.0)	26<br>(8.0)	13<br>(4.0)	11<br>(3.38)	
**D-Dimer**													
Low	8<br>(0.7)	3<br>(37.5)	0<br>(0.0)	0<br>(0.00)	0<br>(0.0)	0<br>(0.0)	2<br>25.00	0<br>(0.0)	0<br>(0.0)	1<br>(12.5)	1<br>(12.5)	1<br>(12.5)	0.3222
High	931<br>(75.5)	245<br>(26.3)	10<br>(1.1)	2<br>(0.2)	54<br>(5.8)	79<br>(8.5)	139<br>(14.9)	166<br>(17.8)	107<br>(11.5)	60<br>(6.4)	27<br>(2.9)	42<br>(4.5)	
Missing	294<br>(23.4)	87<br>29.59	8<br>2.72	1<br>0.34	19<br>6.46	33<br>(11.2)	31<br>(10.5)	47<br>(16.0)	33<br>11.22	20<br>6.80	8<br>2.72	7<br>2.38	
**Lactate dehydrogenase (LDH)**													
Normal	24<br>(2.0)	11<br>(45.8)	0<br>(0.0)	0<br>(0.0)	3<br>(12.50)	2<br>(8.3)	1<br>(4.2)	4<br>(16.7)	1<br>(4.2)	1<br>(4.2)	1<br>(4.2)	0<br>(0.0)	0.4324
High	852<br>(69.1)	227<br>(26.6)	8<br>(0.9)	2<br>(0.2)	51<br>(6.0)	72<br>(8.5)	125<br>(14.7)	154<br>(18.1)	100<br>(11.7)	54<br>(6.3)	22<br>(2.6)	37<br>(4.3)	
Missing	357<br>(29.0)	97<br>(27.2)	10<br>(2.8)	1<br>(0.3)	19<br>(5.3)	38<br>(10.6)	46<br>(12.9)	55<br>(15.4)	39<br>(10.9)	26<br>(7.3)	13<br>(3.6)	13<br>(3.6)	
**Sodium**													
Low	380<br>(30.8)	91<br>(24.0)	5<br>(1.3)	0<br>(0.0)	18<br>(4.7)	32<br>(8.4)	49<br>(12.9)	74<br>(19.5)	56<br>(14.7)	30<br>(7.9)	8<br>(2.1)	17<br>(4.5)	0.375
Normal	654<br>(53.0)	190<br>(29.1)	10<br>(1.53)	3<br>(0.5)	39<br>(6.0)	60<br>(9.2)	97<br>(14.8)	106<br>(16.2)	61<br>(9.3)	40<br>(6.1)	23<br>(3.5)	25<br>(3.8)	
High	47<br>(3.8)	12<br>(25.5)	3<br>(6.4)	0<br>(0.0)	4<br>(8.5)	3<br>(6.4)	6<br>(12.8)	7<br>(14.9)	5<br>(10.6)	3<br>(6.4)	2<br>(4.3)	2<br>(4.3)	
Missing	152<br>(12.3)	42<br>(27.6)	0<br>(0.0)	0<br>(0.0)	12<br>(7.9)	17<br>(11.2)	20<br>(13.2)	26<br>(17.1)	18<br>(11.8)	8<br>(5.3)	3<br>(2.0)	6<br>(4.0)	
**Albumin**													
Low	624<br>(50.6)	149<br>(23.9)	10<br>(1.6)	2<br>(0.3)	43<br>(6.9)	52<br>(8.3)	90<br>(14.4)	112<br>(18.0)	80<br>(12.8)	44<br>(7.1)	16<br>(2.6)	26<br>(4.2)	0.7110
Normal	426<br>(34.6)	127<br>(29.8)	5<br>(1.2)	0<br>(0.0)	23<br>(5.4)	42<br>(9.9)	60<br>(14.1)	72<br>(16.9)	41<br>(9.6)	24<br>(5.6)	14<br>(3.3)	18<br>(4.2)	
Missing	183<br>(14.8)	59<br>(32.2)	3<br>(1.6)	1<br>(0.6)	7<br>(3.8)	18<br>(9.8)	22<br>(12.0)	29<br>(15.9)	19<br>(10.4)	13<br>(7.1)	6<br>(3.3)	6<br>(3.3)	
**Alanine Transaminase (ALT)**													
Normal	849<br>(68.9)	230<br>(27.1)	9<br>(1.1)	2<br>(0.2)	51<br>(6.0)	75<br>(8.8)	113<br>(13.3)	143<br>(16.8)	110<br>(13.0)	56<br>(6.6)	27<br>(3.2)	33<br>(3.9)	0.224
High	229<br>(18.6)	60<br>(26.2)	6<br>(2.6)	0<br>(0.0)	15<br>(6.5)	19<br>(8.3)	44<br>(19.2)	43<br>(18.8)	15<br>(6.6)	12<br>(5.2)	4<br>(1.8)	11<br>(4.8)	
Missing	155<br>(12.6)	45<br>(29.0)	3<br>(1.9)	1<br>(0.7)	7<br>(4.5)	18<br>(11.6)	15<br>(9.7)	27<br>(17.4)	15<br>(9.7)	13<br>(8.4)	5<br>(3.2)	6<br>(3.9)	
**Aspartate aminotransferase (AST)**													
Normal	625	186	6	2	40	57	78	94	76	39	22	25	0.157

	(50.7)	(29.8)	(1.0)	(0.3)	(6.4)	(9.1)	(12.5)	(15.0)	(12.2)	(6.2)	(3.5)	(4.0)	
High	452 (36.7)	104 (3.0)	9 (2.0)	0 (0.0)	26 (5.8)	37 (8.2)	79 (17.5)	91 (20.1)	49 (10.8)	29 (6.4)	9 (2.0)	19 (4.2)	
Missing	156 (12.7)	45 (28.9)	3 (1.9)	1 (0.6)	7 (4.5)	18 (11.5)	15 (9.6)	28 (18.0)	15 (9.6)	13 (8.3)	5 (3.2)	6 (3.9)	
**Platelets**													
Low	205 (16.6)	62 (30.2)	6 (2.9)	1 (0.5)	13 (6.3)	17 (8.3)	28 (13.7)	32 (15.6)	23 (11.2)	16 (7.8)	2 (1.0)	5 (2.4)	0.826
Normal	833 (67.6)	222 (26.7)	10 (1.2)	2 (0.2)	49 (5.9)	74 (8.9)	125 (15.0)	144 (17.3)	93 (11.2)	50 (6.0)	27 (3.2)	37 (4.4)	
High	112 (9.1)	26 (23.2)	1 (0.9)	0 (0.0)	6 (5.4)	13 (11.6)	13 (11.6)	19 (17.0)	14 (12.5)	12 (10.7)	4 (3.6)	4 (3.6)	
Missing	83 (6.7)	25 (30.1)	1 (1.2)	0 (0.0)	5 (6.0)	8 (9.6)	6 (7.2)	18 (21.7)	10 (12.1)	3 (3.6)	3 (3.6)	4 (4.8)	
Total	1233	335 (27.2)	18 (1.5)	3 (0.2)	73 (5.9)	112 (9.1)	172 (14.0)	213 (17.3)	140 (11.4)	81 (6.6)	36 (2.9)	50 (4.1)	
